# Viruses with U-DNA: New Avenues for Biotechnology

**DOI:** 10.3390/v13050875

**Published:** 2021-05-10

**Authors:** Kinga K. Nagy, Mikael Skurnik, Beáta G. Vértessy

**Affiliations:** 1 Department of Applied Biotechnology and Food Sciences, Budapest University of Biotechnology and Economics, 1111 Budapest, Hungary; 2 Research Centre for Natural Sciences, Institute of Enzymology, 1117 Budapest, Hungary; 3 Department of Bacteriology and Immunology, Medicum, Human Microbiome Research Program, Faculty of Medicine, University of Helsinki, 00014 Helsinki, Finland; mikael.skurnik@helsinki.fi; 4 Division of Clinical Microbiology, Helsinki University Hospital, HUSLAB, 00290 Helsinki, Finland

**Keywords:** phages, uracil-DNA, biotechnology

## Abstract

Deoxyuridine in DNA has recently been in the focus of research due to its intriguing roles in several physiological and pathophysiological situations. Although not an orthodox DNA base, uracil may appear in DNA via either cytosine deamination or thymine-replacing incorporations. Since these alterations may induce mutation or may perturb DNA–protein interactions, free living organisms from bacteria to human contain several pathways to counteract uracilation. These efficient and highly specific repair routes uracil-directed excision repair initiated by representative of uracil-DNA glycosylase families. Interestingly, some bacteriophages exist with thymine-lacking uracil-DNA genome. A detailed understanding of the strategy by which such phages can replicate in bacteria where an efficient repair pathway functions for uracil-excision from DNA is expected to reveal novel inhibitors that can also be used for biotechnological applications. Here, we also review the several potential biotechnological applications already implemented based on inhibitors of uracil-excision repair, such as Crispr-base-editing and detection of nascent uracil distribution pattern in complex genomes.

## 1. Uracil-DNA Metabolism

Viruses, including phages, are the most common organisms on Earth. Although viruses do not represent free-living life forms and require a host cell to grow and multiply, still it is of utmost importance for them to preserve the integrity of their genetic information, encoded either in DNA or RNA. For DNA viruses, as for all organisms with a DNA genome, the usual chemical composition of the DNA constitutes the four major Watson–Crick building blocks, with the bases adenine, thymine, guanine, and cytosine. Currently, we exist in a thymine-DNA-based world; therefore, it is generally expected that DNA contains thymine opposed to adenine, and that the thymine analogue uracil base appears only in RNA.

**Figure 1:**
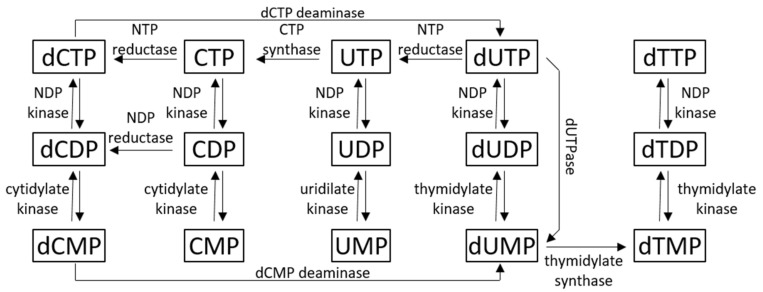
Pyrimidine nucleotide metabolism.

There are multiple routes for the uracil base to appear in DNA, for example, the incorporation of deoxyuridine monophosphate (dUMP) into DNA is one of the most common mistakes in the DNA [1]. The fact that the U-containing DNA (U-DNA) has an increased tendency to generate mutations, has an ability to affect mRNA synthesis, and can cause DNA fragmentation and cell death, demonstrates its biological and evolutionary importance [2]. During evolution, several diverse mechanisms have appeared with responsibility to keep uracil out of DNA. High dUTP concentrations favor misincorporation of U into DNA, and to prevent this event, most cells express dUTPase that is responsible for hydrolyzing dUTP to dUMP [3], thereby providing substrates for thymidylate-synthase to produce dTMP (Figure 1).

Increased incorporation of uracil into DNA takes place mainly when DNA polymerization proceeds under perturbed cellular nucleotide pools and is highly dependent on the cellular dUTP/dTTP ratio. However, cytosine deamination also contributes to genome uracilation. Cytosine deamination can occur either spontaneously or by the activity of enzymes like activation-induced deaminase (AID) and other members of the Apolipoprotein B mRNA editing catalytic polypeptide-like family (APOBEC). The uracil DNA glycosylase enzymes (UDGs) protect the integrity of DNA as they are responsible for the removal of uracil from DNA. The UDG enzyme family can be divided into six subfamilies, summarized below.

### 1.1. Families of Uracil-DNA Glycosylases

**Figure 2:**
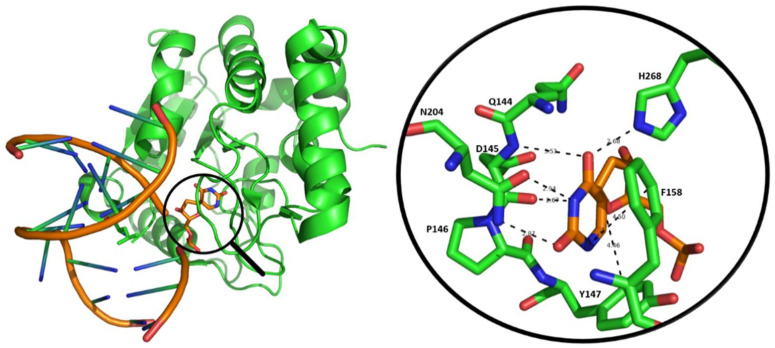
Structure of the UNG protein bound to DNA containing a substrate analogue. On the left: UNG enzyme protein (green ribbon model) binds DNA (orange ribbon model) containing the 2′-deoxy-pseudouridine-5′-monophosphate substrate analogue (stick model, atomic coloring: O: red, N: blue, P, C: orange) (PDB code: 1EMH). On the right: close-up of amino acids (stick model, atomic coloring: O: red, N: blue, C: green) which directly interact with the uracil base (stick model, atomic coloring: O: red, N: blue, P, C: orange).

In Family I, the uracil-DNA *N*-glycosylase enzyme (UNG) is the most significant member of UDGs in uracil removal (Figure 2). UNG also catalyzes the removal of 5-FU (5-Fluorouracil) incorporated into DNA but does not catalyze the cleavage of other 5-substituted uracil derivatives. UNG acts on single- and double stranded DNA as well, with the following preference: ssU > U:G >> U:A [4,5]. Two isoforms of human UNGs are known, the nuclear localized UNG2 and the mitochondrial UNG1. The N-terminus of the nuclear isoform (UNG2) include domains responsible for binding to the single-stranded DNA-binding Replication Protein A (RPA) and the double-stranded DNA-binding Proliferating Cell Nuclear Antigen (PCNA) proteins. RPA and PCNA proteins are crucial factors in replication and the cooperation of UNG with these proteins plays a role in the rapid and efficient repair of replication defects [6].

Family II includes bacterial mismatch-specific uracil DNA glycosylase (MUG) and its homologue in higher organisms, thymine DNA glycosylase (TDG). Both enzymes are active on double-stranded DNA, and both are active in hydrolyzing the N-glycosidic bond of dUMP moieties where the uracil base is located in a mismatch opposite to guanine. These enzymes show no or very weak activity in the case of U:A base pairs. TDG also catalyzes the removal of thymine bases from the T:G defective pairs.

For Family III, the major representative is the single-strand selective monofunctional uracil-DNA glycosylase (SMUG), which is found only in higher eukaryotes. Contrary to its name, it shows greater activity on double-stranded DNA. It removes uracil from DNA both in U:A and U:G base pairs. Members of Families IV to VI are found in thermo- and hyperthermophilic bacteria and Archaea. While all of these enzymes catalyze the removal of uracil from double-stranded DNA, the members of families IV and VI are active on single-stranded DNA as well [5].

No matter which family they belong to, all uracil-DNA glycosylases initiate the base excision repair (BER) pathway, catalyzing the cleavage of the N-glycosidic bond between the base substrate and deoxyribose [7].

### 1.2. Diverse Roles for Uracil in DNA

Although in most cases the presence of uracil is considered as a mistake in the DNA, there are some exceptions where the incorporation of uracil is not damaging or may even be essential. In the case of antibody-producing B lymphocytes, following their antigen-induced activation, a regulated cytosine deamination takes place, catalyzed by AID, a member of the APOBEC enzyme families. Some of the uracil bases thus formed remain unrepaired, resulting in point mutations, and double strand breaks in the DNA during error correction. This mechanism plays an important role in generation of somatic hypermutations as well as in class-switching recombinations, thereby creating diversity in the generated antibodies [8,9].

Other members of the APOBEC enzyme family (mainly members of the APOBEC3 subfamily) also play an important role in immunity towards viruses. It is also essential for viruses to preserve the integrity of their genetic information. The genomes of many viruses (e.g., viruses belonging to the *Herpesviridae*, *Poxviridae*, and *Retroviridae* families) encode proteins (such as dUTPase and/or UNG) that act to minimize genomic uracil levels, thus avoiding mutations. APOBEC proteins catalyze the deamination of cytosine bases in viral DNA to uracil, thereby exerting antiviral activity against several viruses [10,11]. The antiviral effect could be the result of the repair of the resulting U (that may fragment the viral DNA), of the failure of the repair (that results in mutations that impair and prevent virus replication) or, in the case of integrating viruses, the incorporation into the host cell DNA [12]. APOBEC enzyme family members also play an essential role for the proper functioning of the immune system. The AID enzyme is among the most significant APOBEC family members since AID malfunction may lead to the hyper-IgM syndrome. This disease is associated with high IgM levels and a lack of IgG, IgA, and IgE immunoglobulins, making the immune system unable to provide effective protection against viruses and microbes that attack the body [13].

## 2. Intriguing Bacteriophages with Uracil in DNA

Other exceptions in the U substitution can be found among several intriguing bacteriophages, where the viral DNA contains uracil instead of thymine. In these phages, thymine is fully excluded from the DNA. The first such identified viruses with U-DNA were the *Bacillus subtilis* infecting phages PBS1 and PBS2 [14]. In this study, a discrepancy was found between the GC content determined based on its melting temperature and that determined from its buoyant density. In the follow-up experiments to explore this discrepancy using paper chromatography with hydrolyzed phage DNA, thymine base could not be identified. At the same time, the chromatographic and spectrometric measurements suggested that the DNA contained uracil instead of thymine. Furthermore, the GC content (28%) of the PBS2 phage DNA was found to be significantly lower than that of the host DNA (48%), while the base composition of other transducing phages is similar to that of the *Bacillus* hosts [14]. Another U-DNA phage, the *Yersinia* phage ΦR1-37, was isolated 1989 in the Skurnik laboratory from the Turku City sewage [15]. The authors observed aberrant DNA digestion by the restriction enzyme Acc65I, and failed to clone ΦR1-37 DNA into *E. coli* DH10B [16]. This cloning failure in case of DH10B was then found to be due to the presence of U in the phage DNA, since U-containing DNA is known to be degraded by bacterial enzymes such as UNG. During the LC-MS/MS analysis of the hydrolyzed phage DNA, U was identified instead of T. After finding out that T in the DNA was 100% substituted by U, the cloning was successful into *E. coli* CJ236 [*dut*^–^, *ung*^–^] cells that are deficient in uracil-DNA excision repair [16]. The GC content of the ~270 kb phage genomic DNA is 33% while that of the host, *Yersinia enterocolitica* is 47% [17]. ΦR1-37 is the only U-DNA phage with a sequenced and analyzed complete genome. In addition, ΦR1-37 is a member of the so-called “jumbo phages” that have genomes of >200 kb in size. It contains 367 predicted protein coding genes and 5 tRNA genes. A total of 269 of the predicted gene products (73%) lack homologues in sequence databases [17]. 

The identification of U-DNA is complicated by the fact that the sequencing approaches used today do not distinguish between the U and dT bases. To our knowledge, besides PBS1, PBS2 and ΦR1-37, only two other phages are known to carry U-DNA: the *Staphyloccus* phage S6 with a genome size ca. 270 kbp, discovered in 2014 [18] and the *Bacillus subtilis* phage AR9 (with an 251 kbp genome size), isolated in 1968 [19] and sequenced in 2016 [20]. AR9 appears to be very similar to the PBS1 phage and encode a protein with 100% identity to PBS encoded UGI. AR9 also shows some similarities to phage ΦR1-37—both of them are ΦKZ-related phages [20]. However, ΦR1-37 does not encode the UGI protein, originally identified in PBS2 phage and also encoded in AR9. All the known U-DNA phages belong to the family *Myoviridae* (cf Table 1). Members of this phage family possess linear double-stranded genomic DNA [21,22].

From the evolutionary point of view, the bacteria that serve as hosts for the U-DNA phages are found either within the phylum Proteobacteria or the phylum Firmicutes. As the U-DNA phages infect evolutionarily distant bacteria as hosts, it is likely that these phages have independent origins and came into existence through distinct evolutionary events.

The fact that these viruses are able to infect bacteria that contain a highly efficient U-DNA directed repair system to minimize the appearance of U in DNA, is rather thought-provoking. With the exception of Staphylococci that do not have their own dUTPase gene, the host bacteria possess dUTPase, thymidylate synthase, in addition to the ubiquitous UNG, as well. Thus far, it is not fully understood how these phages are able to generate and maintain their U-DNA in the host cell environment, but a drastic reprogramming of the intracellular conditions is certainly needed. To the best of our knowledge, ordinary DNA polymerases use both dUTP and dTTP as substrates during replication, and no extra DNA polymerases have been described in the literature for the host bacteria and for these phages. Therefore, it is likely that the intracellular dUTP/dTTP ratio will determine which nucleotide will be incorporated into DNA versus adenine.

## 3. UNG Inhibitors and Their Potential Biotechnological Applications

The investigation of viruses with U-DNA genomes may be of particular interest as they are unique on Earth. These viruses may constitute an evolutionary relic where DNA has replaced RNA, but uracil from the ancient RNA-based world has not yet been exchanged into thymine. As such, a detailed understanding of U-DNA viruses may provide some insights into early evolutionary events of nucleic acids. Moreover, to survive in bacteria, U-DNA bacteriophages very likely carry genes that encode proteins that can inhibit essential host enzymes such as UNG, dUTPase or thymidylate synthase. In addition to basic academic interest, detailed characterization of these proteins may help to develop new cancer therapies and antibacterial treatments [23]. For example, thymidylate synthase inhibitors, such as 5-fluorouracil (5-FU) or 5-fluorodeoxyuridine (5-FdUrd), are widely used in cancer therapies [24,25,26] and the first dUTPase inhibitors are undergoing detailed investigations [23,27,28,29,30,31,32,33,34]. Inhibition of uracil DNA glycosylase also sensitizes cancer cells to 5-FdUrd similar to like dUTPase inhibition [35]. UNG is also essential for some DNA viruses that infect humans, e.g., herpesviruses, so it has been considered as a target for potential antiviral drug candidates [36]. Below, we focus on the currently identified uracil-DNA glycosylase inhibitors.

### 3.1. Small Inhibitory Molecules

UNG is the main enzyme responsible for uracil repair, and it seems to be essential almost every living organism and some DNA viruses as well. A specific example for UNG importance is observed in, e.g., *Mycobacterium tuberculosis*, where UNG plays a crucial role in maintaining the integrity of its genome especially during in vivo growth because of the exposure of the pathogen to reactive nitrogen intermediates and reactive oxygen intermediates discharges by the host macrophages. These reagents lead to the deamination of cytosine bases of the bacteria GC rich (median GC%: 65.6) genome [37]. In addition, UNG is among the most important factors limiting the efficiency of antifolates and fludarabine. Reduction in UNG activity sensitizes many cancer types to chemotherapy treatment [38]. UNG is also a promising target for drug intervention in protozoan infections, since UNG inhibitors in combination with genotoxic stress effectively suppress the growth of *Plasmodium falciparum*, *Trypanosoma brucei*, and *Trypanosoma cruzi* [38]. In the case of some viruses, UNG is necessary for successful infection. Although HIV is an RNA virus, in the absence of UNG the mutation rate of HIV-1 still shows a drastic increase and renders virus replication inefficient in nondividing cells. Furthermore, the virus particles produced from UNG depleted cells are incapable of infecting new target cells. Thus, there are exciting opportunities for the application of UNG inhibitors as antiretroviral agents as well [39]. Additionally, the crucial role of UNG in DNA repair makes it a very promising pharmacological target in case of cancer therapy and also treatments against pathogens (including bacteria and viruses).

Since UNG seems to be necessary to almost every living organism, it is very important to tailor the specificity of its inhibitors to achieve the required effect. In case of cancer treatment, the inhibitor should be specific to human UNG while not destroying the normal human microbiome in order to minimize side effects. UNG inhibitors against pathogenic microorganisms should specifically target the pathogen. In the specific situation of viral infection of human cells, targeting the human enzyme may still prove to be a good strategy considering that the virus necessary depends on the host enzyme, e.g., in case of a retroviral infection, because the host cell is not as sensitive to UNG inhibition as the virus [39]. The properties of some specific small molecular DNA glycosylase inhibitors are summarized in Table 2.

### 3.2. Protein Inhibitors of Phage Origin

Protein inhibitors are associated with much more higher inhibition efficiency as compared to small molecular UNG inhibitors and of these, the UGI protein (cf. Table 1) is the most effective (with an IC_50_ value of 7.6 pM) [43,44,45]. However, the UGI protein does not show a universal inhibitions against UNG enzymes from any sources, e.g., poxvirus, Vaccinia virus and Mimivirus UNGs are not inhibited by UGI [46]. Still, *E. coli* UNG irreversibly inhibited by UGI [5]. There is a great interest to find new proteinaceous inhibitors as they would make it possible to deeply understand the diverse interaction between UNGs from different species and their inhibitors and help the design of small molecule inhibitors with high specificity and effectivity. In addition, the inhibitors themselves could be used in a number of ways in biotechnology and healthcare.

The first experiments leading to the discovery of an inhibitory protein against UNG were conducted in the Friedberg laboratory after they had observed that the UNG enzyme of *Bacillus subtilis* had lost its activity against U-DNA 4 min after the infection of the bacteria with phage PBS2 [47]. In 1980, the UNG inhibitor (UGI) protein of phage PBS2 was described thus explaining why the phage DNA was not degraded after its entry into the bacterial cell [48]. Z. Wang and D. W. Mosbaugh described a screening method in 1988 to identify the uracil-DNA glycosylase inhibitor in the genome of the PBS2 phage. The *Eco*RI digested phage DNA fragments were ligated into pUC19 vector—thus creating the phage library—and then transformed into *E. coli* KT8052 (*ung*^–^) cells. Significantly higher transformation efficiency was observed for *ung*^–^ cells (8800 transformants per µg of vector DNA) than for *E. coli* expressing the UNG enzyme (250 transformants per µg of vector DNA). M13 phage grown in an *E. coli* (*dut^–^ ung^–^*) strain produced progeny phage that inefficiently propagate in wild type *E. coli* but productively infect *E. coli* (*ung*^–^) strains. Based on these observations, they predicted that M13mpl9 phage containing uracil-DNA would not generally propagate in *E. coli* JM101 carrying the phage genomic fragments containing vectors unless these cells contained a plasmid that expressed functional PBS2 uracil-DNA glycosylase inhibitor. The cells which were supported the productive infection of M13mpl9 phage containing uracil-DNA were used to isolate plasmid DNA which presumably carrying the gene of the potential UNG inhibitor [49].

In recent years, two additional proteins have been identified that also strongly inhibit UNG. The first one in 2006 was the protein p56 of *Bacillus* phage Φ29 [50]. More recently, the UNG inhibitor of *Staphylococcus aureus* was discovered starting from bioinformatic analysis of protein structure data, and based on its origin, was termed as *Staphylococcus aureus* UGI, abbreviated as SaUGI [45,51]. 

Φ29 is a relatively small virus with a genome size of 19 kb encoding only 27 proteins. As a part of the functional characterization of the early viral protein using in vivo chemical cross-linking and affinity chromatography, UNG was identified as the cellular target of the phage Φ29 p56 protein. Interestingly, the UNG-inhibitor p56 is encoded by a non-uracil-DNA containing phage [50]. Phage Φ29 is a podovirus with a linear double-stranded DNA genome of ~19 kb that encodes only 27 proteins. The reason why a non-uracil-DNA phage still needs a UNG-inhibitor may be that the phage replicative intermediates have long stretches of single-stranded DNA that are highly sensitive to degradation. If these single-stranded sections contained U they would be easily degraded by UNG [50,52]. Like UGI, p56 is also an early viral gene product [50,53]. It was demonstrated that p56 forms a DNA-mimicking heterodimer that efficiently inhibits the UNG [44,54].

SaUGI was identified through a bioinformatics analysis that aimed to find potential DNA mimic proteins among previously reported protein structures. Although DNA mimic proteins are essential for living cells and some viruses, only a few DNA mimic proteins (<20) have so far been analyzed in detail. Such proteins are hard to identify because their amino acid sequences and protein structures are extremely divergent and, therefore, a structural homology was used for screening the protein structures deposited in the PDB data base [45].

While the inhibitors UGI and p56 are encoded by bacteriophages, the SaUGI gene is located in a bacterial genome. It is intriguing to understand what physiological role can be attributed to a bacterial UNG inhibitor, since the UNG catalytic action is usually considered to be of key importance in preserving the genome integrity. From this point of view, it is relevant to note that the SaUGI coding gene is actually located on a putatively replicative mobile genetic element within the *Staphylococcus* genome (Staphylococcal Cassette Chromosome (SCC)). Such mobile genetic elements play significant roles in transmission of antibiotic resistance among bacterial strains [55].

**Figure 3:**
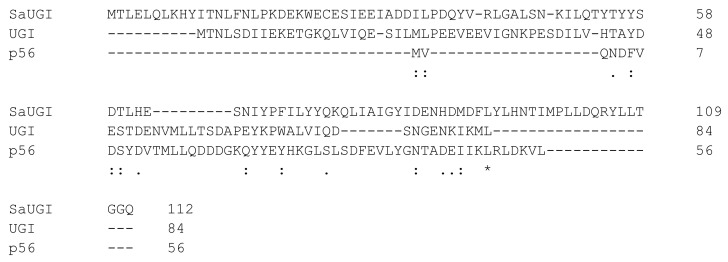
Multiple sequence alignments of three uracil-DNA glycosylase inhibitor proteins. ‘*’ (asterisk) indicates positions which have a single, fully conserved residue; ‘:’ (colon) indicates conservation between groups of strongly similar amino acids; ‘.’ (dot) indicates conservation between groups of weakly similar amino acids.

These three proteins (UGI, p56 and SaUGI) inhibit the members of the same enzyme family, still these proteins do not show any significant sequence similarity to each other—they have clearly been established in distinct evolutionary sequences (Figure 3).

**Figure 4:**
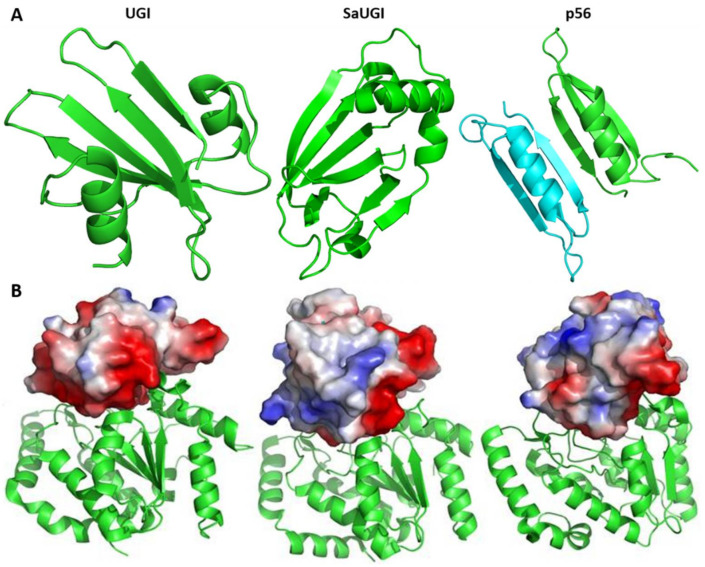
A structural comparison of UGI, SAUGI and p56 and their complexes with UNG. (**A**) Three-dimensional structures of the three known uracil-DNA glycosylase inhibitors, depicted as ribbon models (in green). The two monomers of p56 are shown in different colors (green and cyane). (**B**) Electrostatic representation of three inhibitor proteins (surface models colored according to electrostatics - red symbolizes negative charge, blue positive and white is neutral) in their complexes with UNG (ribbon model). It is clearly visible that in all three cases, the inhibitor protein binds to the UNG DNA-binding groove, mimicking the negative charge of the DNA. The figures were produced using the PyMOL program based on structural data from PDB database (PDB ID: UGI: 1UGI; SaUGI: 3WDG; p56: 2LE2; *E. coli* UNG complex with UGI: 1LQM; *S. aureus* UNG complex with SaUGI: 3WDG; *B. subtilis* UNG complex with p56 dimer: 3ZOQ) [45,54,56,57,58].

Their three-dimensional structure of the UNG inhibitory proteins also revealed that their folding patterns were different (Figure 4). Yet, in any case, they all create a protein surface that mimics DNA and they all bind tightly to UNG.

Besides their clinical significance, the UNG inhibitors could be useful in basic research as well. Here, we present two application examples:

(1) Recently, CRISPR-driven genome engineering has become the gold standard for not only gene destruction, but also for introducing site specific mutations into highly complex genomes. Such CRISPR-based gene editing application required two major modification in the Cas9 nuclease. On the one hand, the nuclease activity was destroyed creating a Cas9 variant that is still capable of site-specific DNA binding but does not cleave the DMS strand. On the other hand, a cytidine deaminase domain (originally present in, e.g., AID) was fused to the catalytically inactive Cas9 protein (Cas9^D10A^). In this fused Cas9^D10A^ construct, the deaminase activity will get constrained to the cytosine base(s) present within the sequence recognition window on the DNA strand. Site specific cytosine deamination leads to a point mutation (C to U, followed by U to T in the next replication cycle). However, this clever system necessarily requires abrogation of uracil directed repair, which is constituted by a further modification of the Cas9^D10A^-deaminase molecular tool. Namely, the uracil DNA glycosylase inhibitor (UGI) is linked to the C-terminus of the Cas9^D10A^-deaminase complex [59].
(2) The appearance of uracil in the DNA, as reviewed above, may report on activation of cytosine-deaminases or spontaneous events of deamination, as well as on dNTP pool perturbations. In all these circumstances the primary effect, i.e., appearance of uracil, is only temporary. Through the highly efficient uracil directed repair, the transient uracil mark is quickly erased, impeding analysis of uracil patterns. To visualize nascent uracils is an important task in order to gain mechanistic insights into the different cellular processes involved in uracil-DNA metabolism. The application of the UNG inhibitor UGI, or other inhibitors with this function is indispensable to decipher uracil patterns [3,60].

## 4. Discussion

We have reviewed some cases that attest to the widespread view of bacteriophages having significant and yet not fully explored potential for biotechnology and biomedicine [61]. Due to the ever increasing occurrence of antibiotic-resistant pathogenic bacteria, the use of bacteriophages to combat bacterial infection (phage therapy) constitutes a research field of high current interest [62]. In addition, the exploitation of the vast genetic pool of bacteriophages in the search of novel possible therapies can serve as key basis for biomedical applications.

An intriguing small group of bacteriophages functions with thymine-less genomic DNA. These phages employ uracil instead of thymine as the adenine-base-pairing constituent in DNA. Although to date there is only a limited number of documented cases of bacteriophages with such uracil-DNA genomes, it has to be mentioned that none of the most widely used DNA sequencing technologies can differentiate between thymine and uracil and, therefore, uracil-DNA sequences escape detection due to this block-out. U-DNA bacteriophages are able to infect bacterial cells in spite of the highly efficient uracil-directed DNA repair pathway that degrades uracil-DNA and, moreover, of the additional mechanisms in pyrimidine metabolism that operate to keep out uracil from DNA via dUTPase and provide dTTP via thymidylate synthase.

Hence, U-DNA bacteriophages can only replicate in the cellular environment if they successfully abolish uracil-DNA attacking mechanisms and also re-program the cell to provide the dUTP building block and its efficient incorporation into phage DNA. Thus far, only one bacteriophage pathway has been identified that counteracts uracil excision from DNA: UNG inhibitor proteins strongly bind to uracil-DNA glycosylase and abolish its catalytic activity [45,48,50]. Biotechnological application of uracil-DNA glycosylase inhibitor allowed fine-tuning of Crispr-Cas-based genome editing [59]. Convergent evolution has led to several differently structured uracil-DNA glycosylase inhibitors, and it is expected that all U-DNA bacteriophages encode such an inhibitor. However, the perturbation of cellular dNTP pool to increase dUTP level is also required to ensure U-DNA synthesis. Since dUTPase and thymidylate synthase enzymes are primarily responsible for decrease in dUTP and increase in dTTP pools, inhibition of these two enzymes may also contribute to the strategy of U-DNA bacteriophages.

Perturbation of dUTP/dTTP pools is also a direct effect of numerous anti-cancer drugs widely used in the clinic (e.g., fluoropyrimidines against thymidylate synthase) [26]. Combined use of thymidylate synthase and dUTPase inhibitors is associated with a synergetic effect in killing cancer cells [28]. A similar increased effect has been observed upon parallel inhibition of UNG and thymidylate synthase [35]. Identification of novel bacteriophage proteins that influence cellular dNTP pools may therefore also contribute to development of novel drugs.

## Figures and Tables

**Table 1:** Uracil-DNA phages.

U-DNA Phages	Family	Produced UNG Inhibitor	Host	Reference
PBS1/PBS2	*Myoviridae*	UGI	*Bacillus* spp.	[14]
ΦR1-37		?	*Yersinia* spp.	[16]
S6		?	*Staphylococcus* spp.	[18]
AR9		UGI	*Bacillus* spp.	[20]

**Table 2:** Properties of some specific DNA glycosylase inhibitors. *: In a follow-up study, several derivatives of this compound were also investigated and showed submicromolar IC_50_ values [39].

Inhibitor Compound	Species	IC_50_	Reference
uracil base	*Mycobacterium tuberculosis*	2.05 mM	[37]
6-(p-n-octylanilino)uracil (OctAU)	*Herpes simplex* virus type1	8 µM	[40]
1-methoxyethyl-6-(p-n-octylanilino)uracil	*Plasmodium falciparum*	16.75 μM	[41]
6-(phenylhydrazino)uracil		77.5 μM	
4-[(1E,7E)-8-(2,6-dioxo-1,2,3,6-tetrahydropyrimidin-4-YL)-3,6-dioxa-2,7-diazaocta-1,7-dien-1-YL]benzoic acid and its derivatives *	human	9 μM	[42]
		0.26 μM	[39]
